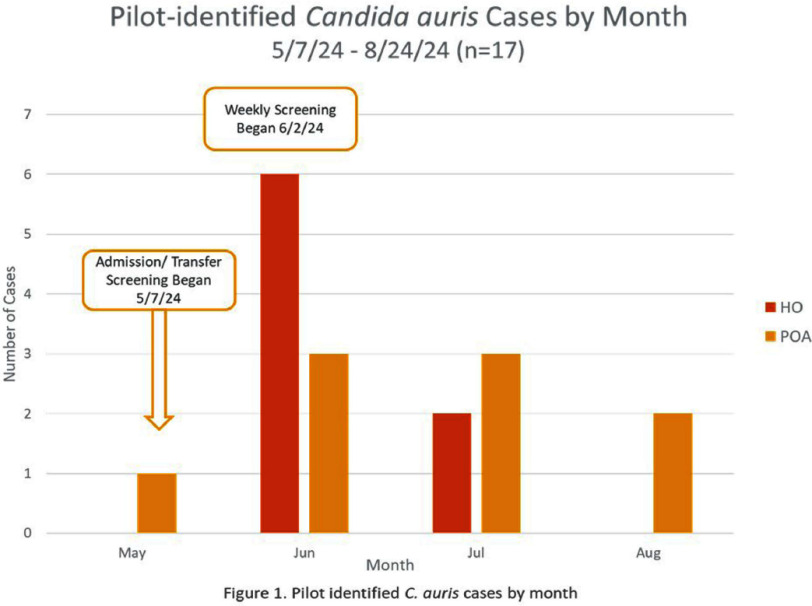# Just Keep Screening: Candida auris Admission and Weekly Surveillance Pilot on High-Risk Units

**DOI:** 10.1017/ash.2025.214

**Published:** 2025-09-24

**Authors:** Rachel Weber, Jacqueline Maniscalco, Grace Barajas, Michael Malczynski, Chao Qi, Teresa Zembower, Maureen Bolon

**Affiliations:** 1 Northwestern Memorial Hospital; 2 Northwestern Medicine; 3 Northwestern University

## Abstract

**Background:** Our Candida auris surveillance protocol dictates that all patients who have been admitted to a skilled nursing facility (SNF), long-term acute care hospital (LTACH), and/or acute inpatient rehab (AIR) in the prior six months be screened on hospital admission. When hospital-onset (HO) cases are identified, point prevalence surveys (PPS) are conducted. Despite this, we identified two units with high prevalence of C. auris and an increasing number of HO cases. To investigate, we initiated an expanded C. auris screening pilot. **Methods:** Infection prevention (IP) verified that two units, the medical intensive care unit (MICU) and the pulmonary medicine unit (PMU) had the highest C. auris prevalence and number of HO cases. We formed a multidisciplinary process improvement team (MPIT) to develop recommendations. A pilot was launched to implement universal admission and transfer screening by PCR and weekly screening by culture on MICU and PMU. Screening consisted of two swabs: bilateral nares and bilateral axilla/groin. For patients with a tracheostomy or endotracheal tube, an endotracheal aspirate was collected. Pilot data were analyzed and shared with executive leadership. **Results:** In the 15 months prior to the pilot, 24/47 (51%) of the hospital-wide HO C. auris cases occurred on the pilot units resulting in 17/40 (43%) of all PPS performed. The pilot, conducted between 5/7/24 – 8/24/24, screened 868 unique patients and detected 9 present-on-admission (POA) C. auris cases and 8 HO C. auris cases (Figure 1). This surveillance avoided a minimum of 7 PPS and identified a cluster of C. auris on MICU. Notably, 9/9 (100%) of the POA cases were exposed to a SNF, LTACH, and/or AIR within 6 months prior to admission. Of the HO cases, 7/8 (88%) were epidemiologically linked with another C. auris patient, and 4/8 (50%) were co-colonized with at least one other multidrug-resistant organism at the time of collection. The pilot was established as routine practice on the two units. **Conclusion:** Our screening pilot identified POA and HO C. auris cases and demonstrated that HO cases decreased over time. This suggests that active surveillance allows for rapid identification and isolation of patients, preventing transmissions and outbreaks. In our experience, IP education and hospital-wide admission screening did not stop cases on units with a high prevalence of patients with C. auris. The pilot confirmed that our current hospital-wide admission screening protocol identifies cases on admission but alone will not prevent nor capture HO cases.

**Figure:**